# Epidemiology, clinical features and outcomes of patients with kidney biopsy proven IgA vasculitis in Auckland and Northland, Aotearoa, New Zealand presenting between 2003–2020.

**DOI:** 10.1186/s12882-025-04362-2

**Published:** 2025-07-31

**Authors:** Janak Rashme de Zoysa, Zhenqiang Wu

**Affiliations:** 1 Renal Service, Health New Zealand Health NZ Te Whatu Ora Waitematā, 122 Shakespeare Road, Takapuna, Auckland, 0622 New Zealand; 2 Department of Medicine, Waitematā Clinical Campus, Faculty of Medicine and Health Sciences, The University of Auckland, Auckland, New Zealand; 3 Epidemiology and Biostatistics, School of Population Health, Faculty of Medicine and Health Sciences, The University of Auckland, Auckland, New Zealand

**Keywords:** Aotearoa New Zealand, Kidney failure, Immunoglobulin A vasculitis, Vasculitis

## Abstract

**Aims:**

Immunoglobulin A vasculitis is a small vessel vasculitis. Here we describe the epidemiology, clinical features, treatment and outcomes in patients, over the age of 16 years, with kidney biopsy proven cases seen in two regions of Aotearoa, New Zealand over an 18-year period.

**Methods:**

Potential cases were identified, in patients over the age of 16 years, who underwent kidney biopsy between 2003 and 2020. A retrospective review of all potential cases was performed.

**Results:**

Thirty-four patients were identified, 10 females and 24 males, of whom 15 were European, 5 Māori, 7 Pacific Peoples, and 7 Asians. The incidence was 1.16 per 1,000,000 patient-years (95% CI: 0.82–1.61), there was an excess in males (RR = 2.42 95% CI 1.16–5.06, *p =* 0.016) with no excess by ethnicity. Mean age at presentation was 42.8 years (range 16.4–70.5). Mean creatinine at presentation was 118µmol/L (range 51–410). Twenty-six patents received corticosteroids, with two patients also receiving cyclophosphamide and five patients receiving azathioprine. Six patients (17.6%) needed long-term kidney replacement therapy, 12 were left with significant chronic kidney disease (35.3%) and 4 died (11.8%) at the end of study follow up. Elevated serum creatinine at presentation was predictive of need for kidney replacement therapy. Age was predictive of mortality.

**Conclusions:**

Immunoglobulin A vasculitis is not overrepresented among indigenous people in Aotearoa New Zealand. The majority of patients receive immunosuppression, however, were left with significant kidney disease.

## Summary at a glance

What is known about this subject?

What does this add?

How this study might affect research, practice or policy

Immunoglobulin A vasculitis (IgAV) is less common in adults than children. The epidemiology and outcomes in adults are poorly described.

IgAV is associated with long-term complications especially kidney disease and has a high mortality.

We highlight a slightly lower incidence of IgAV in adults compared to historic cohorts with no excess by ethnicity. Chronic kidney disease and kidney failure are common and should be monitored for.

Further guidelines and prospective multicentre clinical trials are needed for diagnosis and management of IgAV.

## Introduction

Immunoglobulin A vasculitis (IgAV), previously known as Henoch–Schonlein purpura [1], is an Immunoglobulin A (IgA)-mediated small-sized vessel leukocytoclastic vasculitis characterised by the pentad of: palpable purpura, arthralgia, arthritis, gastrointestinal upset and kidney involvement. IgAV is the most common vasculitis in children where it is typically a self-limited disease [2]. Many criteria have been developed over the years for defining and classifying the disease, including the Paediatric Rheumatology European Society (EULAR/PRINTO/PRES) criteria [3], the American College of Rheumatology (ACR) classification criteria [4], Michel et al. [5] and the Chapel Hill Consensus Conference [1], however, these are for children. In adults, the disease is reported to be less common, with historic data suggesting an annual incidence estimated of 3.4 to 14.3 per 1,000,000 individuals [5, 6] with newer data suggesting the annual incidence in adults varying from 1.5 to 51 per 1,000,000 individuals [7, 8]. Kidney and gastrointestinal involvement are poor prognostic factors and the main cause of morbidity and mortality.

Few studies describe the clinical features and outcomes in adults with kidney biopsy proven disease. Pillebout et al. reported on the largest cohort of 250 IgAV patients from France with an 11% incidence of kidney failure with a further 27% having significant kidney impairment and survival of 74% at a mean follow-up of 14.8 years [9]. Walker et al. described 17 patients from Christchurch, Aotearoa New Zealand (AoNZ), identified between 1973 and 1985, with 18% developing kidney failure and 90% survival at 5 years [10]. Studies from other countries report rates of kidney failure of 15–27% and kidney impairment of 8–31% [11, 12].

Notably the more recent ACR/EULAR endorsed guidelines for vasculitis, as yet, do not have a criterion for IgAV. Treatment guidelines are unclear regarding the role of immunosuppression. Kidney Disease: Improving Global Outcomes (KDIGO) guidelines recommend that glucocorticoid should not be used with isolated extrarenal IgAV and that management should follow the guidance for IgA nephropathy including use of prednisone and cyclophosphamide for patients with crescentic glomerulonephritis with consideration of corticosteroids (CS) for milder cases and the potential for mycophenolate as a steroid sparing agent [13]. The use of rituximab has also been described in case series [14], however, its role is uncertain.

To better understand IgAV, in this study we describe the epidemiology of kidney-biopsy proven IgAV in adult patients from Auckland, and Northland in AoNZ. We also describe the clinical spectrum of presentation, treatments and outcomes and finally evaluate any demographic or clinical associations with the need for kidney replacement therapy (KRT): dialysis or kidney transplantation; or mortality.

## Methods

Ethical approvals were obtained from the Auckland Health Research Ethics Committee (AH25461) and the Waitematā Māori Health Research committee.

The study was undertaken in Northland and Auckland, which encompasses around 37% of the AoNZ population with estimates obtained from Stats New Zealand [15].

All private and public kidney biopsies performed in Auckland and Northland are processed and interpreted at a single laboratory with biopsy samples routinely screened with immunofluorescence and electron microscopy when adequate tissue is available. We note that there is no single diagnostic test for IgAV and that the diagnosis relies on a combination of clinical criteria and laboratory findings. For this study to be considered as having IgAV, patients were to have undergone a kidney biopsy and had have kidney involvement based on histological changes of IgA, namely predominant IgA mesangial deposits and no alternate diagnosis, consistent with the Pillebout classification [9]. In addition, patients were to have the additional presence of one or more of the following: (1) purpura or petechiae with lower limb predominance, (2) abdominal pain, (3) arthritis, or (4) arthralgia. Patients had to present between 1st January 2003 and 31st December 2020 and be 16 years of age or older at the time of first presentation. Patients younger than 16 years at the time of presentation, no kidney biopsy, or no changes of IgA on kidney biopsy or lack additional systemic features consistent with IgAV were excluded.

The clinical records of all potential subjects based on the kidney biopsy features were reviewed. First presentation was defined as when the patient was first seen in a hospital setting. Ethnic group was classified using the prioritisation system in which individuals are allocated to a single ethnic group in an order of priority, if they have identified with more than one group. The order of priority is Māori, then Pacific peoples, Asian, Middle-Eastern/Latin American/African (MELAA), other and finally European [16]. Deprivation was derived by address at time of diagnosis using the validated New Zealand Index of Socioeconomic Deprivation (NZDep) presented as a decile scale from 1 (least deprived) to 10 (most deprived) [17]. Additional data from the community laboratory, primary practice or private tertiary care were obtained. Data was censored for 31st December 2022. Using a Health New Zealand database de-identified population controls from the Northland and Auckland region were risk set matched 1:5 on age, sex and prioritised ethnicity providing that controls remained alive at index, where index was defined as the date of IgAV diagnosis; and without a diagnosis of IgAV. Each matched control was given the same exposure date as their respective IgAV case.

### Statistical analysis

Analysis was performed using SPSS Statistics Version 29. Descriptive statistics were presented as mean ± standard deviation (SD) or median (range), as appropriate. Group differences in baseline characteristics were assessed using t-test/non-parametric test for continuous variables and the χ^2^ test or Fisher’s exact test for categorical variables. A two-sided *p* value < 0.05 was considered statistically significant. The 95% confidence intervals (95%CI) for estimated incidence were calculated assuming a Poisson distribution of the observed cases. Univariate survival analysis were conducted using Cox proportional hazards regression for outcomes of KRT and death. The Kaplan-Meier curve was used to compare survival between cases and matched controls.

## Results

Thirty-four patients with IgAV were identified over the study period: 24 males and 10 females. The mean (± SD) age at presentation was 42.8 ± 17.1 years (range 16.4–70.5) (Table 1). There was a total of 318.8 patient years of follow up, with a mean of 9.38 ± 4.97 years (range 0.6–19.4).

**Table 1:** Demographic characteristics of patients with IgAV at presentation

Age (years +/- SD)	42.8 +/- 17.1
Sex n (%)	
Male	24 (70.6)
Female	10 (29.4)
Ethnicity n (%)	
NZ European	12 (35.3)
NZ Māori	5 (14.7)
Samoan	4 (11.8)
Other European	3 (8.3)
Indian	3 (8.3)
Cook Island Māori	2 (5.9)
Chinese	2 (5.9)
Fijian	1 (2.9)
Filipino	1 (2.9)
Singhalese	1 (2.9)
NZ Dep, mean (SD)	6.8 (2.8)
NZ Dep, n (%)	
1–3	5 (14.7)
4–7	13 (38.2)
8–10	16 (47.1)
Smoking Status n (%)	
Current	5 (14.7)
Ex-smoker	10 (29.4)
Never	18 (52.9)
Unknown	1 (2.9)
BMI (kg/m^2^)	
Mean (Range)	30.32 (15.8–46.2)
Number > 30 (%)	12 (35.3)
Co-morbidities n (%)	
Cardiovascular disease	6 (17.4)
Type 2 Diabetes mellitus	8 (23.5)
Hypertension	10 (29.4)
Chronic Obstructive Airways Disease	1 (2.9)

IgAV: Immunoglobulin A vasculitis, SD: standard deviation; NZ Dep: New Zealand Index of Socioeconomic Deprivation, BMI: Body Mass Index

Five patients were NZ Māori, 7 Pacific peoples, 7 Asian and 15 European. It is estimated that over the 18-year study period, for the Northland and Auckland regions there were 29, 232, 728 patient-years including people only over the age of 16 years, of which 50.2% were females, and 11.54% of the population identifying themselves as NZ Māori, 10.52% as Pacific Peoples, 22.01% as Asian, 1.47% as MELAA and 54.46% as European [15].

We estimated an incidence of IgAV of 1.16 per 1,000,000 patient-years (95% CI: 0.82–1.61). The incidence in males was 1.65 per 1,000,000 patient years (95% CI: 1.08–2.42) and in females was 0.68 per 1,000,000 patient years (95% CI: 1.16–1.25). The rate in males was significantly higher than females: RR = 2.42 95% CI 1.16–5.06, *p =* 0.016. The annual incidence in Māori was 1.48 per 1,000, 000 patient years (95% CI 0.54–3.29), 1.76 per 1,000,000 patient years in Pacific peoples (95% CI 0.77–3.48), 1.09 per 1,000,000 patient years in Asians (95% CI 0.48–2.15), and 9.42 per 1,000,000 patient years in Europeans (95% CI: 5.42–15.19). A Chi-squared goodness of fit test demonstrated no excess by ethnicity *X*^*2*^ (4, 34) = 4.8, *p* = 0.31.

### Clinical features

At presentation 33 patients (97.1%) had skin symptoms, 32 (94.1%) had kidney symptoms, 8 (23.5%) had gastrointestinal (GI) symptoms and 11 (32.4%) had joint symptoms.

At presentation, mean (± SD) serum creatinine was 118µmol/L ± 76, 29 patients had proteinuria and 27 patients had haematuria. All patients went on to have a kidney biopsy at some point in time with all patients having positive staining for IgA and 10 patients having crescents. Selected laboratory data at presentation and kidney histology is presented (Table 2).

**Table 2:** Laboratory data from initial presentation, kidney histology and treatment data

Clinical parameter	All patients (*n* = 34)	No KRT (*n* = 27)	KRT (*n* = 7)	Significance (*p* value*)
Haemoglobin (g/L), mean (SD)	132.8 (21.7)	133.3 (27.5)	132.7 (20.5)	0.95
Platelets (x10^9^/L), mean (SD)	296.2 (96.2)	306.6 (96.2)	256.1 (92.0)	0.22
White cell count (x10^9^/L), mean (SD)	9.1 (2.9)	9.2 (3.1)	8.5 (1.7)	0.54
Serum albumin (g/l), mean (SD)	35.6 (6.2)	35.4 (6.4)	36.6 (5.4)	0.65
Serum creatinine (µmol/L), mean (SD)	118.0 (76.0)	107.8 (57.8)	157.3 (122.3)	0.33
Urine red cells, n (%)				0.13
Absent	7 (20.6)	4 (14.8)	3 (42.9)	
Present	27 (79.4)	23 (85.2)	4 (57.1)	
Proteinuria (mg/mmol), mean (SD)	2397.3 (2256.9)	2150.7 (2257.1)	3348.6 (2146.8)	0.22
Proteinuria (mg/mmol), n (%)				1.00
Absent	5 (14.7)	4 (14.8)	1 (14.3)	
Present	29 (85.3)	23 (85.2)	6 (85.7)	
Glomeruli, mean (SD)	16.6 (10.8)	17.4 (11.4)	13.6 (8.3)	0.42
Fractional scarring of tubulointerstitium (%), mean (SD)	11.0 (15.9)	7.5 (10.9)	24.3 (24.6)	0.12
Abnormal arteries, n (%)	12 (35.3)	9 (33.3)	3 (42.9)	0.68
Crescents, n (%)	10 (29.4)	8 (29.6)	2 (28.6)	1.00
**Treatment**				
Any immunosuppressant, n (%)	8 (23.5)	6 (22.2)	2 (28.6)	1.00
Corticosteroids	8 (23.5)	6 (22.2)	2 (28.6)	1.00
Cyclophosphamide	2 (5.9)	2 (7.4)	0 (0.0)	1.00
Azathioprine	5 (14.7)	3 (11.1)	2 (28.6)	0.27

KRT: Kidney replacement therapy; *, for those continuous variables p values are based on parametric t-test and nonparametric tests yielded similar levels of significance

### Treatment

Nine patients had both intravenous and oral CS, 17 had oral CS and 8 patients did not receive any CS. Two patients had cyclophosphamide in addition to CS, five had azathioprine in addition to CS. One patient had only dapsone as immunosuppression.

### Kidney outcomes

**Fig. 1:**
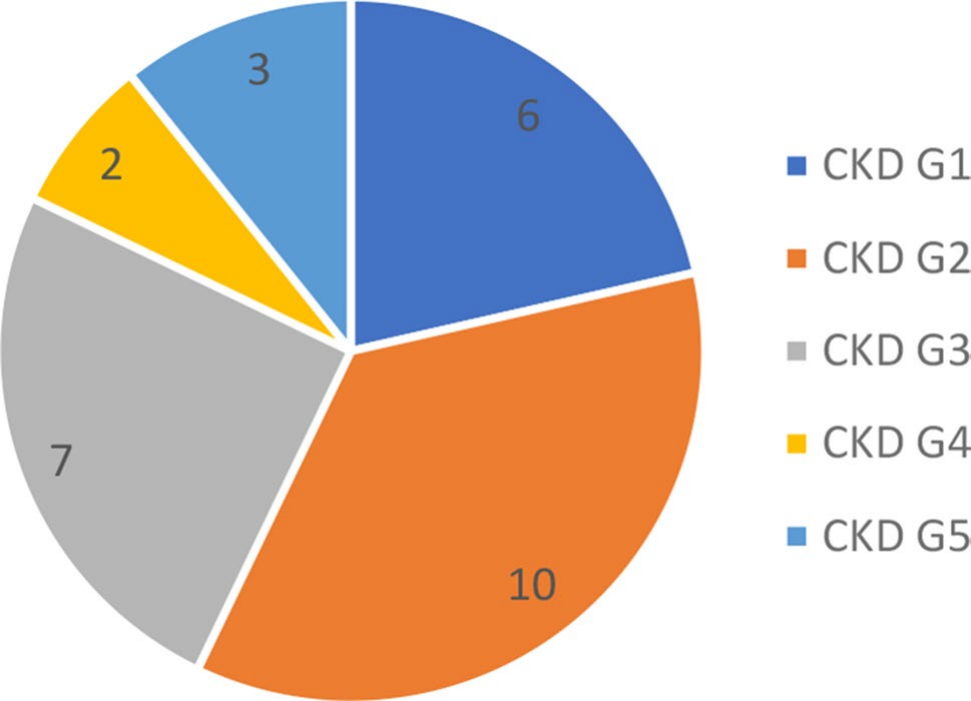
Number of patients with each stage of chronic kidney disease at end of study period, using the three variable Chronic Kidney Disease Epidemiology Collaboration (CKD-EPI) formula. *N* = 28 (patients not on kidney replacement therapy)

One patient needed dialysis acutely and recovered to be independent of KRT to the end of follow up. Six patients developed kidney failure needing chronic KRT; all six had dialysis initially with four patients going on to receive a kidney transplant. Unfortunately, in one patient, the kidney transplant failed, due to recurrence of the primary disease, and required a return to haemodialysis. At last available follow-up in the 28 patients not on KRT the last available mean (± SD) creatinine was 144µmol/L ± 90µmol/L. There were 12 patients left with significant chronic kidney disease (CKD): G3, G4 and G5 (Fig. 1) using the CKD-EPI formula.

Use of any immunosuppressant did not significantly influence the risk of needing KRT (any immunosuppressant *p* = 1.00, CS *p* = 1.00, cyclophosphamide *p* = 1.00, azathioprine *p* = 0.27, Table 2).

### Other outcomes

Eight patients had gastrointestinal (GI) symptoms at presentation, with five reported to have GI bleeding anytime during follow-up, and four patients requiring hospital blood transfusion.

Only one patient had an admission to hospital due to infection while on immunosuppression. Eight patients had diabetes mellitus at the time of diagnosis, one patient developed steroid-induced diabetes which resolved when steroids were halted and two other patients developed diabetes during the study follow-up period. Seven patients reported subsequent cardiovascular events. Two patients had cancer prior to developing IgAV (treated prostate and treated cervical cancer), four patients developed cancer during follow-up.

### Mortality

**Fig. 2:**
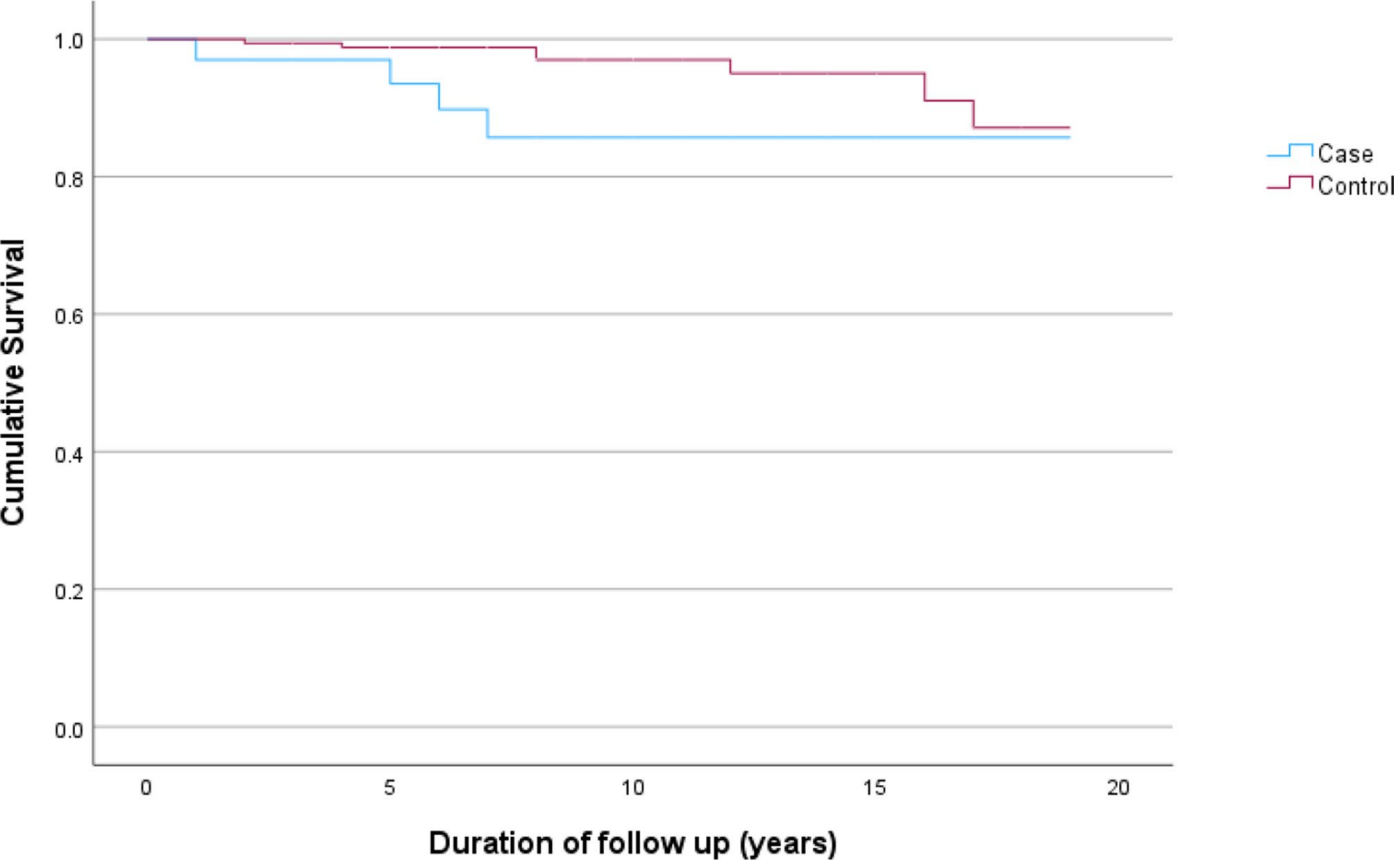
Kaplan Meier survival plot in Immunoglubulin A vasculitis patients compared to age, sex and prioritised ethnicity matched controls (*n* = 170, deaths = 7)

Four patients died (Fig. 2), two of whom were on dialysis representing a cumulative mortality of 11.7%. The cause of death was withdrawal from dialysis, sepsis on dialysis, and for two patients the cause of death was unknown (died at home). In matched controls (*n* = 170; female = 50; mean age 42.8 years; 25 NZ Māori, 35 Pacific peoples, 35 Asian and 75 European) there was no significant difference in mortality (*p* = 0.49).

### Predictors of need for kidney replacement therapy or mortality

We evaluated the selected characteristics of patients who developed the need for KRT or who died with demographic or clinical features at presentation (Table 3). On univariate analysis creatinine at presentation predicted the need for KRT while increased age predicted mortality.

**Table 3:** Univariate survival analysis for KRT and death

Characteristic	Total patients, *n* (column %)	KRT (*n* = 7)		Death (*n* = 4)	
		*n* (row %)	HR (95%CI), *p* value	*n* (row %)	HR (95%CI), *p* value
Age (years), mean (SD)	42.8 (17.1)	38.1 (16.5)	0.99 (0.94, 1.04), 0.60	61.3 (9.7)	1.19 (1.01, 1.39), 0.04
Sex, n (%)					
Male	24 (70.6)	6 (25)	2.27 (0.27, 18.9), 0.45	4 (16.7)	NA
Female	10 (29.4)	1 (10)	1.00 (ref)	0 (0.0)	1.00 (ref)
Ethnic groups, n (%)					
European	15 (44.1)	4 (26.7)	1.00 (ref)	2 (13.3)	1.00 (ref)
Māori	5 (14.7)	1 (20.0)	0.68 (0.08, 6.07), 0.73	1 (20.0)	1.65 (0.15, 18.21), 0.17
Pacific	8 (23.5)	1 (12.5)	0.58 (0.07, 5.25), 0.63	1 (12.5)	1.85 (0.16, 21.59), 0.63
Asian	6 (17.7)	1 (16.7)	0.60 (0.07, 5.37), 0.65	0 (0.0)	NA
Creatinine at presentation (µmol/L), mean (SD)	118.0 (76.0)	157.3 (122.3)	1.01 (1.00, 1.01), 0.04	105.5 (20.1)	1.00 (0.98, 1.01), 0.82
Creatinine at presentation (µmol/L), n (%)					
≤120*	26 (76.5)	5 (19.2)	1.00 (ref)	3 (11.5)	1.00 (ref)
>120*	8 (23.5)	2 (25.0)	2.06 (0.40, 10.8), 0.39	1 (12.5)	1.43 (0.15, 13.77), 0.76
Proteinuria (mg/mmol), median (IQR)	2150 (382, 3910)	2600 (2250, 4630)	NA	1340 (265, 2925)	NA
Proteinuria (mg/mmol), n (%)					
≤500	10 (29.4)	1 (10.0)	1.00 (ref)	2 (20.0)	1.00 (ref)
501–1000	3 (8.8)	0 (0.0)	NA	0 (0.0)	NA
1001–3000	11 (32.4)	3 (27.3)	3.26 (0.34, 31.4), 0.31	1 (9.1)	0.52 (0.05, 5.73), 0.59
>3000	10 (29.4)	3 (30.0)	4.52 (0.47, 43.8), 0.19	1 (10.0)	0.57 (0.05, 6.34), 0.65
Diabetes, n (%)					
Yes	8 (23.5)	3 (37.5)	2.07 (0.46, 9.28), 0.34	3 (62.5)	8.94 (0.92, 86.75), 0.06
No	26 (76.5)	4 (15.4)	1.00 (ref)	1 (3.9)	1.00 (ref)
Smoking status, n (%)					
Current	5 (14.7)	2 (40.0)	1.00 (ref)	1 (20.0)	1.00 (ref)
Ex-smoker	10 (29.4)	2 (20.0)	0.47 (0.07, 3.35), 0.45	2 (20.0)	1.46 (0.12, 17.19), 0.76
Never	18 (52.9)	3 (16.7)	0.40 (0.07, 2.40), 0.32	1 (5.6)	0.30 (0.02, 4.81), 0.40
Unknown	1 (2.9)	0 (0.0)	NA	0 (0.0)	NA
NZ Dep, mean (SD)	6.8 (2.8)	7.1 (3.3)	1.09 (0.81, 1.46), 0.56	7.5 (3.0)	1.16 (0.78, 1.74), 0.46
NZ Dep, n (%)					
1–3	5 (14.7)	1 (20.0)	1.00 (ref)	1 (20.0)	1.00 (ref)
4–7	13 (38.2)	2 (15.4)	0.74 (0.07, 8.22), 0.81	0 (0.0)	NA
8–10	16 (47.1)	4 (25.0)	1.45 (0.16, 13.02), 0.74	3 (18.8)	1.15 (0.12, 11.09), 0.90
Immunosuppression, n (%)					
Yes	26 (76.5)	5 (19.2)	1.00 (ref)	2 (7.7)	1.00 (ref)
No	8 (23.5)	2 (25.0)	1.09 (0.21, 5.63), 0.92	2 (25.0)	2.88 (0.40, 20.46), 0.29

KRT: kidney replacement therapy; HR: hazard ratio from univariate Cox regression; 95%CI, 95% confidence interval; NA, not available; ref, reference group, NZ Dep: New Zealand Index of Socioeconomic Deprivation. * Serum creatinine of < 120 µmol/L is reported as normal at our laboratory

## Discussion

In this series from AoNZ, we saw an incidence of kidney biopsy proven IgAV of 1.16 per 1,000,000 patient-years less than the incidence of historic hospital-based cohorts in adults [15–16]. The incidence is similar to more recent report from Southern Spain [7] and southern Sweden [18] (1.5 and 3.0 per 1,000,000 patient years respectively) but much less than the rate from the UK [19] and Slovenia [8] (22 and 51 per 1,000,000 patient years respectively). The UK study extracted data from an electronic primary practice database while the Solvenia study used a combination of ICD codes and a search of IgAV compatible biopsy records. These search strategies are likely to contribute to increased identification. In addition, the interaction of environment and genetics, specific to AoNZ may potentially have a role.

An increased incidence in males has been previously reported in both adults [18, 20, 21] and children [20, 21] and was seen in our series. Importantly, we saw no difference in the incidence of IgAV between ethnic groups in NZ. Two studies in children have highlighted difference in incidence by ethnicity [22, 23]. However, these differences have not been reported in adult studies. Even controlling for age and diabetes NZ Māori, the indigenous people of NZ, are noted to have 1.56-fold higher risk and Pacific people to have a 2.86-fold higher risk of CKD compared to non-Maori, non-Pacific people [24] and an increased rate of some glomerulonephritidies [25] are seen NZ by ethnic group. However, in this series no difference was seen for IgAV.

All patients with suspected IgAV should have their kidney function monitored. In adults there are no clear recommendation on when a kidney biopsy should be performed [26], however, in general the presence of kidney impairment, haematuria or proteinuria > 1gm/day is an indication for kidney biopsy. Kidney disease is reported to be more common and more severe in adults with IgAV than in children [27]. We found 17.6% of patients in our cohort needed chronic KRT, with a further 35.2% being left with CKD. This is substantially higher than that reported for the general AoNZ population [24, 28] but similar to rates seen in other adult series of IgAV [9–12].

Observational data suggests that CS are effective in managing symptoms of arthritis and GI discomfort and CS and/or dapsone can be used for skin disease. In our series the majority of patients (76.5%) received CS with a fifth of patients (20.6%) receiving concurrent therapy with azathioprine or cyclophosphamide. We found that the use of these medications did not impact on the future need for KRT. Notably, calcineurin inhibitors, mycophenolate, nor rituximab were used for any patient in our cohort possibly reflecting funding for these medication in AoNZ and lack of robust evidence for their use. Although cyclophosphamide has been used for many forms of vasculitis, in the only study in IgAV, Pillebout et al. compared cyclophosphamide and CS versus CS alone and found no difference between the two groups [29]. No trial data explores the use of Azathioprine in adults and limited data in the use of other immunosuppressants. Hernández-Rodriguez performed a systematic review of rituximab in IgAV and identified 35 paediatric and adult patients with refractory or resistant disease to steroids and found rituximab was useful in inducing remission in 74.3% of cases [14]. It should be noted that we had small numbers of patients and did not have robust histology to guide treatment decisions. In patients with deteriorating renal function with proliferative changes or cellular crescents on kidney biopsy where no irreversible changes are present aggressive immunosuppression should be considered.

GI disease is reported to occur in up to half of patients depending on the case series [27]. In our cohort 23.5% of patients reported GI symptoms at presentation, none required laparotomy, and 14.7% had GI bleeding at any time.

Mortality was high; four patients died (11.8%) during study follow up. However, when comparing to a matched cohort we did not see an increase in mortality. This is out of keeping with other studies [18, 27].

Unsurprisingly, on univariate analysis, an elevated creatinine at presentation was associated with an increased risk of KRT while increased age was associated with mortality. Multivariable Cox regression was not conducted due to the small sample size, and the lack of other significant associations observed in the univariate analyses. Criteria for kidney histology to determine prognosis have been undertaken in IgAV with the International Study of Kidney Disease in Children system the best known [30], however this is only relevant in children. More recently the Oxford MEST-C classification (mesangial proliferation, endothelial proliferation, sclerosis, tubulointerstitial atrophy and fibrosis and crescents) has been shown to be useful in determining prognosis in children and adults with IgAV [31]. Unfortunately, this scoring system was not available for the majority of biopsy samples and was not able to be assessed in this study.

This study is likely to underreport the incidence of IgAV. Not all cases of IgAV may proceed to kidney biopsy and this study would miss any such cases. Notably, in the series from Hočevar et al. who report an incidence of 51 per 1,000,000 adults, of 81 patients, 79 had skin biopsies and 2 had skin and kidney biopsies [8]. The criteria used to define IgAV may impact on the incidence. Watts et al. look at a cohort in adults and noted that if using the ACR criteria the incidence of IgAV (reported as Henoch–Schonlein purpura) was 1.3 per 100,000 but in the same cohort using the CHCC definition the incidence was 0.34 per 100,000 [6]. We used the EULAR/PRINTO/PRES criteria, with kidney involvement as an a priori criteria, which has been noted to have high sensitivity and specificity compared to other criteria [32]. Although this study encompasses a long time period, from four districts of AoNZ, the small number of subjects may impact on the incidence reported and should be interpreted with caution.

All patients included in this series were biopsy proven thus preventing incorrect diagnosis. Few studies report on adult IgAV. Walker et al. report on the clinical outcomes of a AoNZ cohort but not the epidemiology [10]. The NZ glomerulonephritis study reporting on patients undergoing kidney biopsy between 1972 and 1983 combined patients with IgA and IgAV [33]. Thus, to the best of our knowledge, this is the first report of the epidemiology of IgAV from AoNZ.

In summary, this retrospective cohort study demonstrated an incidence of kidney biopsy proven IgAV of 1.16 per 1, 000,000 patient-years with an excess in males but no excess by ethnicity. We saw most patients were treated with CS with some use of second-line immunosuppression with no use of mycophenolate nor rituximab. We see a high incidence of kidney disease but no increase in mortality.

## Data Availability

Upon reasonable request, deidentified data may be obtained from the corresponding author subject to ethical approval.
